# eQuilibrator 3.0: a database solution for thermodynamic constant estimation

**DOI:** 10.1093/nar/gkab1106

**Published:** 2021-11-29

**Authors:** Moritz E Beber, Mattia G Gollub, Dana Mozaffari, Kevin M Shebek, Avi I Flamholz, Ron Milo, Elad Noor

**Affiliations:** Novo Nordisk Foundation Center for Biosustainability, Technical University of Denmark, Kemitorvet, 2800 Kongens Lyngby, Denmark; Unseen Biometrics ApS, Fruebjergvej 3, 2100 København Ø, Denmark; Department of Biosystems Science and Engineering and SIB Swiss Institute of Bioinformatics, ETH Zürich, Basel 4058, Switzerland; Department of Biosystems Science and Engineering and SIB Swiss Institute of Bioinformatics, ETH Zürich, Basel 4058, Switzerland; Institute of Chemical Sciences and Engineering, EPFL, Lausanne 1015, Switzerland; Department of Chemical and Biological Engineering, Chemistry of Life Processes Institute, and Center for Synthetic Biology, Northwestern University, Evanston, IL 60208, USA; Division of Biology and Biological Engineering, California Institute of Technology, Pasadena, CA 91125, USA; Department of Plant and Environmental Sciences, Weizmann Institute of Science, Herzl 234, Rehovot 7610001, Israel; Department of Plant and Environmental Sciences, Weizmann Institute of Science, Herzl 234, Rehovot 7610001, Israel

## Abstract

eQuilibrator (equilibrator.weizmann.ac.il) is a database of biochemical equilibrium constants and Gibbs free energies, originally designed as a web-based interface. While the website now counts around 1,000 distinct monthly users, its design could not accommodate larger compound databases and it lacked a scalable Application Programming Interface (API) for integration into other tools developed by the systems biology community. Here, we report on the recent updates to the database as well as the addition of a new Python-based interface to eQuilibrator that adds many new features such as a 100-fold larger compound database, the ability to add novel compounds, improvements in speed and memory use, and correction for Mg^2+^ ion concentrations. Moreover, the new interface can compute the covariance matrix of the uncertainty between estimates, for which we show the advantages and describe the application in metabolic modelling. We foresee that these improvements will make thermodynamic modelling more accessible and facilitate the integration of eQuilibrator into other software platforms.

## INTRODUCTION

The field of thermodynamics started in the midst of the industrial revolution as an effort to improve mechanical engines (1). The phenomenal success of the theory to describe the relationships between *energy*, *heat*, and *work* and to provide accurate predictions of what is feasible, inspired countless other scientific endeavors, including molecular dynamics and even economics (2–4). Curiously, until recently, thermodynamic reasoning was relatively underutilized in metabolic modelling. We have identified four major reasons for this. The knowledge gap—equilibrium constants for most biochemical reactions have not been measured. The computation gap—thermodynamic constraints tend to make metabolic models more complicated. For example, Flux Balance Analysis (FBA) with thermodynamic constraints turns from a standard Linear Problem to an NP-complete Mixed-Integer one (MILP) (5,6). The motivation gap—it is not clear to everyone that using thermodynamics in models is actually necessary or even useful. The accessibility gap—adding thermodynamics to an existing model is laborious. It involves tasks such as: mapping identifiers, adjusting the Δ*G*′° values to the aqueous conditions, and annotating charged molecules correctly—to name but a few.

One of the major breakthroughs in bridging the knowledge gap was suggested by Lydersen *et al.* (7), who adapted the *group contribution* method (which can computationally predict molecular Gibbs energies) to organic chemistry. Decades later, Joback *et al.* (8) and Mavrovouniotis *et al.* (9) eventually implemented this idea for the context of biochemical reactions. This data-driven approach decomposes a compound into a list of predefined chemical groups, each of which is assumed to contribute a fixed amount to the compounds’ Gibbs energy of formation. These group contributions are estimated by regression against measurements of reaction Gibbs energies, thus enabling estimation of thermodynamic potentials for the majority of reactions in central carbon metabolism (10).

In the last decade, several improvements to the accuracy and coverage of this method have been proposed and implemented (11–14). Nevertheless, some estimates cannot be performed using group contributions since certain molecular structures still do not have any experimental data and therefore reactions in which they appear are outside the scope of the methods. Furthermore, some structures seem to violate the assumption of independence between groups and tend to greatly increase the error of the estimates. In the end, although increasing accuracy by improving the method itself or by gathering more experimentally-derived equilibrium constants is still definitely worthwhile, one could argue that the current state-of-the-art has reached a sufficient level for many applications.

Similarly, the complexity gap has changed from a hard barrier to a minor inconvenience. Increasingly faster computers and powerful MILP solvers (such as IBM CPLEX and Gurobi which offer free academic licenses) made it possible to solve large problems using personal computers, a task which was inconceivable only a decade ago.

The motivation gap is harder to overcome due to a chicken-and-egg problem. Since other technical issues delayed the application of thermodynamic models, it was difficult to demonstrate the usefulness of these models and therefore convince the scientific community that they are worth investing in. Nevertheless, several methods that take advantage of thermodynamic data already exist. Some exploit thermodynamic principles to constrain reaction directionality and metabolite concentrations (5,15,16). Others use thermodynamic driving forces as a proxy for pathway efficiency (17,18). More recently, probabilistic methods combining thermodynamic parameters have been suggested for parameter estimation (19) and flux sampling (20). These algorithms have the potential to improve the flux predictions produced by flux analysis (21–23), and assist in the design of new metabolic pathways (18). However, one of the toughest barriers computational approaches need to overcome is gaining the trust of experimental biologists, and it appears that a few cracks are forming already. For instance, thermodynamic driving forces were used to show how glycolysis is passively regulated (24), and predictions based on thermodynamic enzyme efficiency (25) have proven to fit quite well with experimental data from *Z. mobilis* (26).

It seems that the time has come to address the accessibility gap. In recent years, a plethora of software tools have facilitated the reconstruction, validation, and analysis of genome-scale metabolic models and made them a community standard which is applied in thousands of scientific projects every year (27). In 2012, we launched the first version of the eQuilibrator website, which aimed to broaden access to thermodynamic parameters and thermodynamic reasoning about metabolism (28). eQuilibrator provides a simple search-focused interface for quickly finding the Gibbs energy change of a biochemical reaction, and is now used by ∼1000 distinct users every month. However, eQuilibrator was designed to be used for single reactions. Therefore, it is inefficient at querying lists of reactions and does not account for correlations between multiple estimates. In this paper, we present a new Python package which is aimed at both novice and expert programmers that want to add thermodynamic parameters to their models.

## RESULTS

The Python package equilibrator-api, which is freely available on the Python Package Index and on conda-forge, significantly lowers the barrier to thermodynamic modelling in multiple use-cases. In fact, some of the thermodynamic analyses mentioned in the previous section have used an early version of this package to obtain the necessary thermodynamic constants. For example, a recent study (22) tested the hypothesis that an upper limit exists on the total Gibbs energy dissipation rate of cellular metabolism using estimates generated by equilibrator-api. Since this analysis required standard Gibbs energy estimates for thousands of reactions, this would have been untractable using the web interface. Genome-scale metabolic models with annotated metabolites, that use any of the standard chemical identifiers contained in MetaNetX, can make use of dedicated functions that provide a mapping to eQuilibrator compounds with thermodynamic information. These compounds can then be used by constraint-based and sampling frameworks, such as multiTFA (6) and Probabilistic Thermodynamic Analysis (PTA) (20). In the case of specific pathway models, such as ones designed for metabolic engineering and contain novel reactions, our framework provides additional tools for assessing the feasibility of the pathway (17,18).

Furthermore, since the launch of eQuilibrator in 2012 (28) we added a list of new features: a hundred times larger compound database, the ability to locally add new compounds to the database, calculating the full covariance matrix for the uncertainty between estimates, support for multi-compartment reactions, changing magnesium ion concentrations, tools for analysing whole pathways, and general improvements in speed and memory use. Moreover, we now base all estimates on the component contribution method (13), which required developing a way to calculate component contribution estimates for new reactions on-the-fly. The basis for these calculations is provided in the supplementary section S1. Please note, that some of these features are not yet accessible through the online version of eQuilibrator, namely the larger database, adding new compounds, the covariance matrix, and multi-compartment reactions.

Below, we expand on some of these new features, and the benefits they provide to our users.

### Covariance matrix

One of the advantages provided by the new system is the ability to compute the covariance matrix for the uncertainty of multiple estimates (a feature currently supported only in the Python package). In some cases, standard transformed formation (Δ_*f*_*G*′°) or reaction (Δ_*r*_*G*′°) energy estimates have large uncertainties when taken individually. However, uncertainties might be highly correlated, e.g., when reactions share a common compound or compounds share a common chemical group. Ignoring these correlations usually overestimates the uncertainty and may violate the first law of thermodynamics (13). In contrast to per-reaction and per-compound uncertainties, the covariance matrix describes the joint uncertainty precisely.

The general formula for the standard Gibbs energy of a set of reactions, given their mean $\boldsymbol{\mu }$ and covariance $\mathbf {\Sigma }$ is

(1) $$\begin{equation*} \Delta _r G^{\prime \circ }= \boldsymbol{\mu } + \mathbf {\Sigma }^{1/2}\mathbf {m}\;, \end{equation*}$$

where $\mathbf {\Sigma }^{1/2}$ is a square root of $\mathbf {\Sigma }$. $\mathbf {m}$ can either be a standard multivariate normal random variable in case of random sampling applications or an optimization variable bounded within a desired confidence level for constraint-based approaches. Importantly, this formulation ensures that the computed Gibbs energies are consistent with the first law of thermodynamics, relieving the need for computationally more challenging constraints such as Gibbs energy balance (22) (see supplementary section S2 for more details).

This formulation of the uncertainty has already been available in a development version of eQuilibrator for some time and was successfully applied in both statistical and constraint-based contexts: In PTA (20) it is used to detect thermodynamic inaccuracies in metabolic models, to predict occurrences of substrate channeling, as well as to estimate metabolite concentrations and reaction fluxes with a sampling algorithm. Moreover, multiTFA (6) shows that accounting for covariance reduces the ranges of reaction energies also in constraint-based models.

### Expanding the scope of compounds

**Figure 1.**
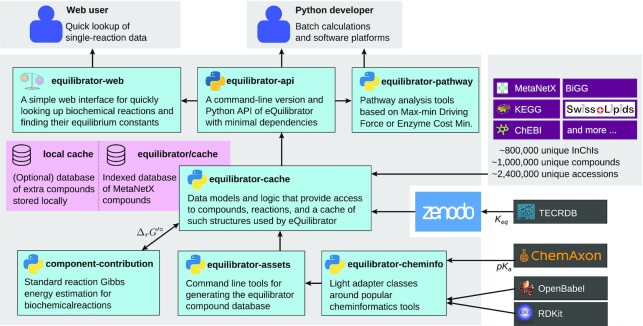
The design of the eQuilibrator 3.0 suite. At the core, the equilibrator-cache Python package defines and manages the database of all compounds, denoted *equilibrator/cache*. All compound identifiers and names come from MetaNetX, which aggregates several popular compound repositories. The component-contribution package is responsible for handling training data, group decomposition, Legendre transforms (for pH, pMg and ionic strength adjustments), and the final estimation of Gibbs energies for new compounds and reactions. Some scripts required for rebuilding the database and adding new compounds to a local database are stored in equilibrator-assets. All data relevant for running the eQuilibrator packages is stored in Zenodo and is freely available. This includes the experimental $K_{{\rm eq}}$ data used to train the component contribution method, which comes mainly from NIST TECR database (46). The equilibrator-api package exposes nearly all functions relevant for users with sufficient programming skills, and is required for running eQuilibrator in batch mode (e.g. on an entire metabolic model). Some advanced features (namely pathway profiling using Max–min Driving Force analysis (17) or Enzyme Cost Minimization (47)) are available through a separate package called equilibrator-pahtway. For non-programmers, the web interface provides quick and easy access to eQuilibrator estimates, but is only designed to deal with a single reaction at a time.

A frequent request from eQuilibrator users was adding compounds that are not present in the KEGG database (29). We therefore modified eQuilibrator to use MetaNetX (30), a database that aggregates chemicals that are relevant for metabolic models from multiple online databases, including KEGG (29), ChEBI (31), BiGG(32), ModelSEED(33), Swiss Lipids(34) (see Figure 1). This expanded the repertoire from ∼10 000 to ∼1 000 000 compounds which can be accessed using identifiers from various namespaces. As a result, eQuilibrator can now be used with metabolic models from different sources (e.g., SEED or BiGG models) directly, without the need to map all compounds to KEGG identifiers in advance.

The eQuilibrator website was originally designed to provide pre-calculated Gibbs energy values for a large database of biochemical reactions. Advanced users often want to calculate Δ*G* values for molecules and reactions not found in the database (even after the expansion to MetaNetX). For example, metabolic pathway engineering often utilizes promiscuous enzymes to generate novel reactions, producing pathways with compounds not found in MetaNetX (35–39).

Previously, this would have required users to add their compounds to the database and rerun the component-contribution method – a considerable effort. To reduce the amount of time it takes to estimate Δ*G* values of reactions with novel compounds we have extended the equilibrator-assets package, which is responsible for generating the compound database, to enable users to directly add new entries to a local version of the compound database (i.e. a *local cache* as depicted in Figure 1). The new compounds can be specified using the IUPAC International Chemical Identifier (InChI) or the Simplified Molecular Input Line Entry System (SMILES). These compounds can then be used directly with the equilibrator-api package, allowing for seamless integration of new compounds in thermodynamic analyses (40). See the documentation at https://equilibrator.readthedocs.io for more details.

### Multi-compartment reactions

The standard calculation for reaction Gibbs energies assumes that all reactants are in the same aqueous compartment, with a constant pH, pMg, ionic strength and temperature (41). However, most genome-scale metabolic models describe more than one compartment, usually separated by a lipid membrane, and contain many transport reactions that span compartments with different aqueous conditions. Furthermore, the membrane between each two compartments can be associated with an electrostatic potential ΔΦ (in units of volts) which affects the thermodynamics of charged ions travelling between them. When a reaction involves transport of metabolite species between compartments with different hydrogen ion activity or electric potential, we add the following term to its Δ_*r*_*G*′°:

(2) $$\begin{equation*} -N_H \cdot RT\ln \left( 10^{\Delta \mathrm{pH}} \right) - Q \cdot F \Delta \Phi \end{equation*}$$

where *R* is the gas constant, *T* is the temperature, *F* is Faraday’s constant (the total electric charge of one mole of electrons –96.5 kC mol^−1^), ΔpH is the difference in pH between initial and final compartment, *N*_H_ is the net number of hydrogen ions transported from initial to final compartment, and *Q* is the stoichiometric coefficient of the transported charges (41,42). Note that *RT*ln (10^ΔpH^) is the energy difference between the compartments specifically for protons (due to their concentration gradient), and *F*ΔΦ is the energy difference relevant to all charges (including protons).

In prior versions of eQuilibrator, adjusting the standard Gibbs energy of multi-compartment biochemical reactions, such as ones facilitated by membrane transporters, had to be performed as a post-processing step. For example, the *vonBertalanffy* extension in the COBRA toolbox (43) performs such additional calculations as part of its pipeline. In eQuilibrator 3.0, we add functions to facilitate the adjustments required for multi-compartment reactions as part of our Python package. See supplementary section S3 for an example.

### Adjusting estimates to different pMg values

Since ions are abundant in the cytosol and can bind metabolites to varying degrees, ion concentrations have a large effect on biochemical thermodynamics (41). The concentration of protons (H^+^), commonly expressed as the pH, is the most dramatic example of this phenomenon. However, this is not the only case. Magnesium ions (Mg^2 +^) bind to many common biochemical moieties, especially phosphate, and have been shown to play a significant role in the thermodynamics of glycolysis (44). For example, the dissociation constants for ATP and ADP are low enough for them to be in their complex forms Mg · ATP^2 −^ and Mg · ADP^−^ at a physiological intracellular pMg of 3 (which is defined similarly to pH, i.e. it corresponds to a concentration of 10^−3^ M—or 1 mM—of Mg^2 +^).

Every compound can be seen as an ensemble of *pseudoisomers*, molecules only differing in protonation or magnesium binding state. In a biochemical context, where all compounds are assumed to be in a buffered aqueous environment, we do not distinguish between pseudoisomers and refer to the entire ensemble as a *metabolite*. Note that this assumption does not hold for transport reactions across membranes, which may selectively transport only specific pseudoisomers. For single compartment reactions it is thus convenient to discuss the standard *transformed* Gibbs energy of formation Δ_*f*_*G*′° which groups together all pseudoisomers into one formation energy. It can be obtained using a Boltzmann-weighted mixture of its constituent pseudoisomers:

(3) $$\begin{equation*} \Delta _f G^{\prime \circ } = -RT\ln {\sum _j\mathrm{e}^{-\Delta _f G^{\prime \circ }(j)/(RT)}}. \end{equation*}$$

The standard transformed Gibbs energies of formation Δ_*f*_*G*′°(*j*) for each pseudoisomer *j* at given biochemical conditions can be calculated using the Legendre transformation

(4) $$\begin{equation*} \Delta _f G^{\prime \circ }(j) = \Delta _f G^\circ (j) + \Theta _H(j) + \Theta _{Mg}(j), \end{equation*}$$

where

$$\begin{eqnarray*} \Theta _H(j) &\equiv& - N_H(j)[\Delta _f G^\circ (H^+) + RT\ln (10^{-pH})] \\ \Theta _{Mg}(j) &\equiv& - N_{Mg}(j)[\Delta _f G^\circ (Mg^{2+}) + RT\ln (10^{-pMg})]. \end{eqnarray*}$$

The first term—Δ_*f*_*G*°(*j*)—is the *chemical* standard Gibbs energy of formation of the pseudoisomer. The second term—Θ_*H*_(*j*)—describes the contribution of protons to the Gibbs energy as a function of the pH. *N*_*H*_(*j*) is the number of protons associated to this pseudoisomer, and Δ_*f*_*G*°(*H*^+^) is the standard formation energy of a proton, defined to be 0 in this framework (41). Similarly, the effect of the concentration of Mg^2 +^ ions (quantified as pMg) can be taken into account by adding the term Θ_*Mg*_(*j*). Similar to the case of protons, *N*_*Mg*_(*j*) is the number of Magnesium ions associated to this pseudoisomer, and Δ_*f*_*G*°(*Mg*^2 +^) is the standard formation energy of Magnesium ions, equal to −455.3 kJ/mol.

Affinity to Mg^2 +^ varies between compounds and pseudoisomers. The presence of certain chemical moieties, such as phosphate groups, tends to increase the binding affinity (45), while increasing the protonation state tends to decrease the affinity. Unfortunately, the availability of affinity constants for Mg^2 +^ is much lower than for H^+^. In eQuilibrator, we used Δ_*f*_*G*′°(*j*) for magnesium-bound pseudoisomers collected by Vojinović and von Stockar (44) and affinities predicted by Du *et al.* (14). For all other pseudoisomers, we assumed that their affinity is weak and has negligible effect on thermodynamics.

After populating the database with magnesium-bound pseudoisomers, we computed the root mean square error (RMSE) for all reactions from the NIST TECR database (46). When taking magnesium into account, the RMSE improved slightly from 2.99 to 2.93.

In the online version of eQuilibrator, pMg can be adjusted in the same way as pH and ionic strength, by entering the value in the proper text field. Similarly, the Python package contains a new p_mg variable that can be set directly, along with p_h and ionic_strength, as explained in the documentation at https://equilibrator.readthedocs.io.

### Upgrades to the eQuilibrator website

Compared to its original version (28), the eQuilibrator website has undergone several major updates. As mentioned earlier, the backend method for estimating reaction Gibbs energies was changed to component contribution (13). This method provides a consistent set of reaction energies, without compromising the accuracy of reactions and compounds with direct experimental data. More recently, we added the ability to adjust the pMg as well as an option to plot Δ*G*′ as a function of pMg. A more comprehensive list of changes can be found on the website itself at https://equilibrator.weizmann.ac.il/static/classic_rxns/updates.html.

Besides improvements to the core search function, the website now offers tools for pathway analysis based on two methods: Max-min Driving Force (MDF) (17) and Enzyme Cost Minimization (47). Both these methods aim to provide a quantitative scoring system that can be used to rank natural or engineered metabolic pathways – either to assist in the design process for metabolic engineers, or for understanding the design principles that govern the evolution of metabolic pathways. MDF is a purely thermodynamic-based approach that assumes driving forces are maximized within the constraints imposed by metabolite concentration bounds and the necessary coupling between adjacent reactions. ECM uses a convex optimization approach to find the optimal distribution of enzyme and metabolite concentrations, where the pathway flux is maximized. Both algorithms are also available in the Python package named equilibrator-pathway.

### Complete code refactoring

In order to extend the eQuilibrator framework to new uses-cases (such as those described above) a complete code refactoring was required—creating separate packages for each distinct function. The original code was designed exclusively for a single workflow: starting with analysing the chemical structures (group decompositions), reverse-transforming the measured equilibrium constants to chemical Gibbs energies (48), solving the linear regression problems to find the group contribution energies, and using the solutions to estimate formation energies for all compounds in the KEGG database. This long procedure was not useful for users who only wanted to apply component-contribution on a list of their own reactions.

Therefore, we have redesigned the entire component-contribution package, moved it to a new Git repository, and integrated it completely into a larger framework denoted *eQuilibrator 3.0 suite* (see Figure 1). In addition, we raised the coding standards, e.g. by running automated tools for coverage, unit-testing, and documentation (available on equilibrator.readthedocs.io). We also facilitated the installation of the packages by submitting them to the Python Package Index (https://pypi.org) and conda-forge (https://conda-forge.org).

Following this refactoring, Python users can easily install the equilibrator packages on their own computer using a simple command (with full support for Windows OS, MacOS, and Linux). The improved documentation and software stability facilitate this further, even for novice programmers. Furthermore, proper versioning enables researchers to easily trace back changes and reproduce their results generated by older versions of the software.

## DISCUSSION

The eQuilibrator 3.0 suite marks a major shift in the focus of our software which has so far been mainly geared for single reaction searches or small biochemical networks (pathways) and exposed via a web interface. Now, we reach out to a larger audience, including modellers who want to populate genome-scale metabolic networks with thermodynamic parameters as well as metabolic engineers who want to scan a large set of parameters for their designs. In addition, the software package can now be more easily integrated into other Python-based frameworks and pipelines such as COBRApy (49,50), MEMOTE (51), ModelSEED (52) and CarveMe (53). Furthermore, in supplementary section S1.4, we show how one can efficiently store a set of pre-calculated matrices that can be used to calculate the final estimates (including full uncertainty matrices). This can facilitate building compound databases for frameworks that are not based on Python, or that rely on mathematical calculations that are not yet supported within the eQuilibrator codebase.

The major improvements that we introduce in this work are: (i) an API supported by a refactored codebase that is more suited to modelling applications and integration into other software, (ii) improvements in speed and memory use, (iii) correction for Mg^2 +^ ions, (iv) multi-compartment reactions, (v) access to the full covariance matrix for uncertainty modelling, (vi) cross-databases identification of molecules and reactions with a larger pool of compounds (provided by MetaNetX) and the ability to add novel compounds. We continue to support community-driven development and open source standards, by publishing all the code under the permissive MIT license and making it available on GitLab. All other raw data needed for the algorithm is licensed under a Creative Commons 4.0 license, and stored on www.zenodo.org.

We are open to suggestions for what could be added to eQuilibrator in future via discussions in GitLab issues and we welcome contributions from the community. For example, new features already under consideration include temperature adjustment based on separate entropy/enthalpy estimates, a fully automated script for populating SBML (Systems Biology Markup Language) models with thermodynamic parameters (including multi-compartment reactions), and integration with common platforms and use-cases such as support for Thermodynamic-based Flux Analysis (16) in COBRApy.

We believe that eQuilibrator 3.0 is a substantial step forward in closing the accessibility gap and hope that together with other recent advances (16,17,19,20,47) will bring forth the golden age of thermodynamics in the field of metabolic modelling.

## DATA AVAILABILITY

The source code for all the Python packages mentioned in this manuscript can be found at https://gitlab.com/equilibrator. All relevant data and models are stored on Zenodo, and are linked to from the repository (Training data - https://doi.org/10.5281/zenodo.3978439; Compound database - https://doi.org/10.5281/zenodo.4128542; Model parameters - https://doi.org/10.5281/zenodo.4013788; Group definitions - https://doi.org/10.5281/zenodo.4010929). The documentation can be found at https://equilibrator.readthedocs.io.

The eQuilibrator suite depends on several open-source packages, such as: OpenBabel (54), RDKit, NumPy, SciPy, Pandas, Pint, and SQLAlchemy.

In addition, estimating acid-base dissociation constants was done using Marvin Calculator, Calculator version 21.13.0, ChemAxon (www.chemaxon.com), under an academic license. Marvin Calculator is also required for adding new compounds to a local database.

## Supplementary Material

gkab1106_Supplemental_File
