# Supplementary material for: eQuilibrator 3.0: a database solution for thermodynamic constant estimation
Source: Nucleic Acids Res. 2021 Nov 29;50(D1):D603–9. doi: 10.1093/nar/gkab1106 (PMC8728285; doi:10.1093/nar/gkab1106)
Supplement: gkab1106_Supplemental_File [file gkab1106_supplemental_file.pdf]

Supplementary Text: eQuilibrator 3.0 – a database solution for thermodynamic constant estimation

Moritz E. Beber[1,2,†], Mattia G. Gollub[3], Dana Mozaffari[3,4], Kevin M. Shebek[5], Avi Flamholz[6], Ron Milo[7], and Elad Noor[7,†,*]

[1]Novo Nordisk Foundation Center for Biosustainability, Technical University of Denmark, Kemitorvet, 2800 Kongens Lyngby, Denmark
[2]Unseen Biometrics ApS, Fruebjergvej 3, 2100 København Ø, Denmark
[3]Department of Biosystems Science and Engineering and SIB Swiss Institute of Bioinformatics, ETH Zürich, Basel, 4058, Switzerland

[4]Institute of Chemical Sciences and Engineering, EPFL, Lausanne, 1015, Switzerland
[5]Department of Chemical and Biological Engineering, Chemistry of Life Processes Institute, and Center for Synthetic Biology, Northwestern University, Evanston, Illinois 60208, USA
[6]Division of Biology and Biological Engineering, California Institute of Technology, Pasadena, CA 91125, USA
[7]Department of Plant and Environmental Sciences, Weizmann Institute of Science, Rehovot, Israel

[†]These authors contributed equally.
[*]Corresponding author. Email: elad.noor@weizmann.ac.il

October 18, 2021

S1 Fast calculation of Gibbs energies using component contributions

It is a specific challenge to use the Component Contribution (CC) method in a website such as eQuilibrator, since uncertainty calculations must be made on-the-fly, but the methodology for CC was developed as a one-step calculation for thousands of reactions in a model. The time needed to run CC even for a single reaction is too long to be useful for a website, even if it can be optimized and decreased to a few seconds.

Therefore, we introduced a pre-processing step in which relatively small intermediate matrices are stored in-memory and are used for fast, on-the-fly calculations. This approach provides a trade-off between memory requirements and calculation time.

We begin, in subsection S1.1, with a short summary of the results presented in the original component contribution paper [6]. Then, subsection S1.2 describes the full derivation of the one-the-fly method for the mean estimate values and the uncertainties. In order to facilitate the implementation of this method by others, we summarize only the essential part for the implementation in subsection S1.3. Finally, in subsection S1.4 we describe a corollary of this method, which is a way to maintain a database of the pre-processed information for a very large list of compounds, while requiring relatively small amounts of memory.

S1.1 Standard Component Contribution

According to the standard CC method [6], the vector of estimated standard Gibbs energies for a set of target reactions is given by the formula

$$\boldsymbol{\Delta_r G^\circ}(\mathbf{X}) = \mathbf{X}^\top \left[\underbrace{\mathbf{P}_{\mathcal{R}(\mathbf{S})} \left(\mathbf{S}^\top\right)^+}_{\text{RC}} + \underbrace{\mathbf{P}_{\mathcal{N}(\mathbf{S}^\top)} \mathbf{G} \left(\mathbf{S}^\top \mathbf{G}\right)^+}_{\text{GC}} \right] \boldsymbol{\Delta_r G^\circ}_{obs} \ , \tag{1}$$

where $\mathbf{X}$ is the stoichiometric matrix of the set of target reactions, $\mathbf{S}$ is the stoichiometric matrix of the observed training data, $\boldsymbol{\Delta_r G^\circ}_{obs}$ is a vector of the observed standard Gibbs energies, $\mathbf{G}$ is the group incidence matrix, and $\mathbf{P}_{\mathcal{R}(\mathbf{S})}$ and $\mathbf{P}_{\mathcal{N}(\mathbf{S}^\top)}$ are the orthogonal projection matrices onto the range of $\mathbf{S}$ and the nullspace of $\mathbf{S}^\top$, respectively. As indicated by the braces under the two parts, we can separate the contributions to two: Reactant Contributions (RC) and Group Contributions (GC).

The covariance matrix of the uncertainty in these Gibbs energies is given by:

$$\boldsymbol{\Sigma}(\mathbf{X}) = \mathbf{X}^\top \left[\alpha_{rc}^2 \cdot \mathbf{C}_{rc} + \alpha_{gc}^2 \cdot \mathbf{C}_{gc} + \alpha_{\infty}^2 \cdot \mathbf{C}_{\infty} \right] \mathbf{X} \tag{2}$$

where

$$\begin{aligned}
\alpha_{rc} &\equiv \frac{||\mathbf{e}_{rc}||}{\sqrt{n - \text{rank}(\mathbf{S})}} \\
\alpha_{gc} &\equiv \frac{||\mathbf{e}_{gc}||}{\sqrt{n - \text{rank}(\mathbf{S}^\top \mathbf{G})}} \\
\mathbf{C}_{rc} &\equiv \mathbf{P}_{\mathcal{R}(\mathbf{S})} \left(\mathbf{S}\mathbf{S}^\top\right)^+ \mathbf{P}_{\mathcal{R}(\mathbf{S})} \\
\mathbf{C}_{gc} &\equiv \mathbf{P}_{\mathcal{N}(\mathbf{S}^\top)} \mathbf{G} \left(\mathbf{G}^\top \mathbf{S}\mathbf{S}^\top \mathbf{G}\right)^+ \mathbf{G}^\top \mathbf{P}_{\mathcal{N}(\mathbf{S}^\top)} \\
\mathbf{C}_{\infty} &\equiv \mathbf{G} \mathbf{P}_{\mathcal{N}(\mathbf{S}^\top \mathbf{G})} \mathbf{G}^\top
\end{aligned} \tag{3}$$

and $\mathbf{e}_{rc}$ and $\mathbf{e}_{gc}$ are the residuals of the reactant and group contribution regressions, and n is the number of columns in S.

Note that the diagonal values in $\boldsymbol{\Sigma}(\mathbf{X})$ are the *squared* standard errors of the estimates of single reactions.

S1.2 The on-the-fly Component Contribution method

S1.2.1 Calculating Gibbs energy estimates on-the-fly

What happens when we want to estimate the Gibbs energy of reactions with reactants that are not in $\mathbf{S}$? The long way would be to augment $\mathbf{S}$, $\mathbf{G}$ and $\mathbf{X}$ with more rows that would correspond to the new compounds. Note that if we do not have a group decomposition of one of these new compounds, there is no way to make the estimation (we cannot add "group columns" like we did for compounds in the training set). Fortunately, we will soon see that the effect of the added rows on the calculation is minimal, and it is easy to do the pre-processing trick we need.

Let $\mathbf{G}'$ be the group incidence matrix of only the new compounds, and $\mathbf{X}'$ the sub-matrix of $\mathbf{X}$ corresponding to the new compounds. Then the new matrices we need to use for CC are:

$$\bar{\mathbf{X}} \equiv \begin{bmatrix} \mathbf{X} \\ \mathbf{X}' \end{bmatrix}$$
$$\bar{\mathbf{S}} \equiv \begin{bmatrix} \mathbf{S} \\ \mathbf{0} \end{bmatrix} \tag{4}$$
$$\bar{\mathbf{G}} \equiv \begin{bmatrix} \mathbf{G} \\ \mathbf{G}' \end{bmatrix}$$

We can see that $\bar{\mathbf{S}}^\top \bar{\mathbf{G}} = \mathbf{S}^\top \mathbf{G}$. Since we added only zeros to $\mathbf{S}$, the range will not change, and the null-space of $\mathbf{S}^\top$ will include all the new rows. Therefore

$$\begin{aligned} \mathbf{P}_{\mathcal{R}(\mathbf{S})} &\equiv \begin{bmatrix} \mathbf{P}_{\mathcal{R}(\mathbf{S})} & \mathbf{0} \\ \mathbf{0} & \mathbf{0} \end{bmatrix} \\ \mathbf{P}_{\mathcal{N}(\mathbf{S}^\top)} &\equiv \begin{bmatrix} \mathbf{P}_{\mathcal{N}(\mathbf{S}^\top)} & \mathbf{0} \\ \mathbf{0} & \mathbf{I} \end{bmatrix} \end{aligned} \tag{5}$$

So, the RC term will not change at all, while the GC term can be rewritten in block-matrix form:

$$\begin{aligned} \bar{\mathbf{X}}^\top \mathbf{P}_{\mathcal{N}(\mathbf{S}^\top)} \bar{\mathbf{G}} &= \begin{bmatrix} \mathbf{X} \\ \mathbf{X}' \end{bmatrix}^\top \begin{bmatrix} \mathbf{P}_{\mathcal{N}(\mathbf{S}^\top)} & \mathbf{0} \\ \mathbf{0} & \mathbf{I} \end{bmatrix} \begin{bmatrix} \mathbf{G} \\ \mathbf{G}' \end{bmatrix} \\ &= \begin{bmatrix} \mathbf{X}^\top \mathbf{P}_{\mathcal{N}(\mathbf{S}^\top)} \mathbf{G} \\ \mathbf{X}'^\top \mathbf{G}' \end{bmatrix} \end{aligned}$$

Therefore, when we combine both RC and GC, the upper blocks will sum up to the same value of $\mathbf{\Delta_r G}^\circ(\mathbf{X})$ before we added $\mathbf{X}'$, and the bottom blocks will add a new term, namely:

$$\mathbf{\Delta_r G}^\circ(\bar{\mathbf{X}}) = \mathbf{\Delta_r G}^\circ(\mathbf{X}) + \mathbf{X}'^\top \mathbf{G}' \left(\mathbf{S}^\top \mathbf{G}\right)^+ \mathbf{\Delta_r G}^\circ{}_{obs} \tag{6}$$

Finally, we can define the pre-processing vectors (which depend only on the training data and not on the target reactions we wish to estimate) as:

$$\begin{aligned} \mathbf{v}_r &\equiv \left[\mathbf{P}_{\mathcal{R}(\mathbf{S})} \left(\mathbf{S}^\top\right)^+ + \mathbf{P}_{\mathcal{N}(\mathbf{S}^\top)} \mathbf{G} \left(\mathbf{S}^\top \mathbf{G}\right)^+\right] \mathbf{\Delta_r G}^\circ{}_{obs} \\ \mathbf{v}_g &\equiv \left(\mathbf{S}^\top \mathbf{G}\right)^+ \mathbf{\Delta_r G}^\circ{}_{obs} \end{aligned} \tag{7}$$

and get that

$$\mathbf{\Delta_r G}^\circ(\bar{\mathbf{X}}) = \mathbf{X}^\top \mathbf{v}_r \ + \ \mathbf{X}'^\top \mathbf{G}' \mathbf{v}_g \tag{8}$$

S1.2.2 Calculating uncertainty estimates on-the-fly

If we look again at the definitions in section S1.1, we can see that $\bar{\mathbf{C}}_{rc} = \mathbf{C}_{rc}$ is not affected by the new compounds in X', besides some zero-padding for adjusting its size. For simplicity, we define the term $\mathbf{\Gamma} \equiv (\mathbf{G}^\top \mathbf{S} \mathbf{S}^\top \mathbf{G})^+$, which is a symmetric matrix containing group covariances among the reactions in $\mathbf{S}$. From what we saw in the previous section, $\bar{\mathbf{S}}^\top \bar{\mathbf{G}} = \mathbf{S}^\top \mathbf{G}$, and therefore $\bar{\mathbf{\Gamma}} = \mathbf{\Gamma}$. We can then write:

$$\begin{aligned} \bar{\mathbf{C}}_{gc} &= \mathbf{P}_{\mathcal{N}(\mathbf{S}^\top)} \bar{\mathbf{G}} \left(\bar{\mathbf{G}}^\top \bar{\mathbf{S}} \bar{\mathbf{S}}^\top \bar{\mathbf{G}}\right)^+ \bar{\mathbf{G}}^\top \mathbf{P}_{\mathcal{N}(\mathbf{S}^\top)} \\ &= \begin{bmatrix} \mathbf{P}_{\mathcal{N}(\mathbf{S}^\top)} \mathbf{G} \\ \mathbf{G}' \end{bmatrix} \mathbf{\Gamma} \begin{bmatrix} \mathbf{P}_{\mathcal{N}(\mathbf{S}^\top)} \mathbf{G} \\ \mathbf{G}' \end{bmatrix}^\top \\ &= \begin{bmatrix} \mathbf{C}_{gc} & \mathbf{P}_{\mathcal{N}(\mathbf{S}^\top)} \mathbf{G} \mathbf{\Gamma} \mathbf{G}'^\top \\ \mathbf{G}' \mathbf{\Gamma} \mathbf{G}^\top \mathbf{P}_{\mathcal{N}(\mathbf{S}^\top)} & \mathbf{G}' \mathbf{\Gamma} \mathbf{G}'^\top \end{bmatrix} \end{aligned} \tag{9}$$

and the third term in equation (2) will change to:

$$\begin{aligned}\bar{\mathbf{C}}_\infty &= \begin{bmatrix}\mathbf{G}\\ \mathbf{G}'\end{bmatrix} \mathbf{P}_{\mathcal{N}(\mathbf{S}^\top\mathbf{G})} \begin{bmatrix}\mathbf{G}\\ \mathbf{G}'\end{bmatrix}^\top \\ &= \begin{bmatrix}\mathbf{C}_\infty & \mathbf{G}\mathbf{P}_{\mathcal{N}(\mathbf{S}^\top\mathbf{G})}\mathbf{G}'^\top \\ \mathbf{G}'\mathbf{P}_{\mathcal{N}(\mathbf{S}^\top\mathbf{G})}\mathbf{G}^\top & \mathbf{G}'\mathbf{P}_{\mathcal{N}(\mathbf{S}^\top\mathbf{G})}\mathbf{G}'^\top\end{bmatrix}\end{aligned} \tag{10}$$

Finally, combining these results in the formula for the new uncertainty, we get:

$$\begin{aligned}\mathbf{\Sigma}(\mathbf{X}) &= \bar{\mathbf{X}}^\top \left(\alpha_{rc}\cdot\bar{\mathbf{C}}_{rc} + \alpha_{gc}\cdot\bar{\mathbf{C}}_{gc} + \infty\cdot\bar{\mathbf{C}}_\infty\right)\bar{\mathbf{X}} \\ &= \begin{bmatrix}\mathbf{X}^\top & \mathbf{X}'^\top\mathbf{G}'\end{bmatrix} \begin{bmatrix}\mathbf{C}_1 & \mathbf{C}_2\\ \mathbf{C}_2^\top & \mathbf{C}_3\end{bmatrix} \begin{bmatrix}\mathbf{X}\\ \mathbf{G}'^\top\mathbf{X}'\end{bmatrix}\end{aligned} \tag{11}$$

where we define:

$$\begin{aligned}\mathbf{C}_1 &= \alpha_{rc}\cdot\mathbf{C}_{rc} + \alpha_{gc}\cdot\mathbf{C}_{gc} + \alpha_\infty\cdot\mathbf{C}_\infty \\ \mathbf{C}_2 &= \alpha_{gc}\cdot\mathbf{P}_{\mathcal{N}(\mathbf{S}^\top)}\mathbf{G}\mathbf{\Gamma} + \alpha_\infty\cdot\mathbf{G}\mathbf{P}_{\mathcal{N}(\mathbf{S}^\top\mathbf{G})} \\ \mathbf{C}_3 &= \alpha_{gc}\cdot\mathbf{\Gamma} + \alpha_\infty\cdot\mathbf{P}_{\mathcal{N}(\mathbf{S}^\top\mathbf{G})}\,.\end{aligned} \tag{12}$$

As can be seen in section S2, it is typically more convenient to use the square root of the covariance. As a Hermitian positive-definite matrix, $\mathbf{\Sigma}(\mathbf{X})$ can be decomposed using the Cholesky method and therefore $\exists\mathbf{M}\in\mathbb{R}^{n\times q}$ such that $\mathbf{M}$ is lower diagonal with positive diagonal values, and $\mathbf{M}\mathbf{M}^\top = \mathbf{\Sigma}(\mathbf{X})$. q is the rank of $\mathbf{\Sigma}(\mathbf{X})$.

In practice, however, the Cholesky decomposition often fails due to numerical issues, especially when $\mathbf{\Sigma}(\mathbf{X})$ is very large. Here, we present a different approach which takes advantage of the inner structure of $\mathbf{\Sigma}(\mathbf{X})$.

We start by defining the matrix $\mathbf{L}$:

$$\mathbf{L} \equiv \begin{bmatrix}\alpha_{rc}\ \mathbf{S}^+\mathbf{P}_{\mathcal{R}(\mathbf{S})} & \mathbf{0} \\ \alpha_{gc}\ (\mathbf{G}^\top\mathbf{S})^+\mathbf{G}^\top\mathbf{P}_{\mathcal{N}(\mathbf{S}^\top)} & \alpha_{gc}\ (\mathbf{G}^\top\mathbf{S})^+ \\ \alpha_\infty\ \mathbf{P}_{\mathcal{N}(\mathbf{S}^\top\mathbf{G})}\mathbf{G}^\top & \alpha_\infty\ \mathbf{P}_{\mathcal{N}(\mathbf{S}^\top\mathbf{G})}\end{bmatrix}^\top \tag{13}$$

and one can convince oneself that:

$$\mathbf{L}\mathbf{L}^\top = \begin{bmatrix}\mathbf{C}_1 & \mathbf{C}_2\\ \mathbf{C}_2^\top & \mathbf{C}_3\end{bmatrix}\,.$$

The problem is that $\mathbf{L}$ is extremely rank deficient, which means that it contains a lot of linearly dependent columns (and therefore far from a compact representation of the square root). We can thus perform a rank revealing QR factorization [1] in order to eliminate these redundant columns and end up with a compact matrix $\mathbf{L}_q$, such that $\mathbf{L}_q\mathbf{L}_q^\top = \mathbf{L}\mathbf{L}^\top$. This is effectively equivalent to the Cholesky decomposition of the (constant) inner part of $\mathbf{\Sigma}(\mathbf{X})$.

In the case of the eQuilibrator training database, the number of columns in $\mathbf{L}_q$ is $q = 669$, and the number of rows is $N_c + N_g$ – or about 800. Therefore, amount of memory required to store this matrix is only about 2MB.

Note, that $\mathbf{L}_q$ can be calculated in a pre-processing phase even before we decide on the $\bar{\mathbf{X}}$ matrix and which new compounds are needed (represented by $\mathbf{X}'$ and $\mathbf{G}'$). The only post-processing step left is to define:

$$\mathbf{Q} = \bar{\mathbf{X}}^\top \begin{bmatrix}\mathbf{I} & \mathbf{0}\\ \mathbf{0} & \mathbf{G}'\end{bmatrix}\mathbf{L}_q \tag{14}$$

and as we will see, the covariance matrix will simply be given by $\mathbf{Q}\mathbf{Q}^\top$:

$$\begin{aligned} \mathbf{Q}\mathbf{Q}^\top &= \begin{bmatrix}\mathbf{X}^\top & \mathbf{X}'^\top\end{bmatrix} \begin{bmatrix}\mathbf{I} & \mathbf{0} \\ \mathbf{0} & \mathbf{G}'\end{bmatrix} \mathbf{L}_q \mathbf{L}_q^\top \begin{bmatrix}\mathbf{I} & \mathbf{0} \\ \mathbf{0} & \mathbf{G}'^\top\end{bmatrix} \begin{bmatrix}\mathbf{X} \\ \mathbf{X}'\end{bmatrix} \\ &= \begin{bmatrix}\mathbf{X}^\top & \mathbf{X}'^\top\mathbf{G}'\end{bmatrix} \begin{bmatrix}\mathbf{C}_1 & \mathbf{C}_2 \\ \mathbf{C}_2^\top & \mathbf{C}_3\end{bmatrix} \begin{bmatrix}\mathbf{X} \\ \mathbf{G}'^\top\mathbf{X}'\end{bmatrix} \\ &= \boldsymbol{\Sigma}(\mathbf{X}) \end{aligned} \tag{15}$$

S1.3 Essential information for implementing the on-the-fly Component Contribution method

Here we provide the essentials for implementing the component contribution so that the Gibbs energy and uncertainties of a new set of reactions (not included in the original set) can be estimated on-the-fly. We seperate the process into two steps: the *pre-processing step* (which is rather fast, but can take a few minutes depending on the size of the database) and the *estimation step* (which should take much less than one second).

S1.3.1 Pre-processing step

In the pre-processing step, we calculate the $\boldsymbol{\mu}$ vector, which can be seen as the formation energies of reactants and groups:

$$\boldsymbol{\mu} \equiv \begin{bmatrix} \mathbf{P}_{\mathcal{R}(\mathbf{S})} (\mathbf{S}^\top)^+ + \mathbf{P}_{\mathcal{N}(\mathbf{S}^\top)} \mathbf{G} (\mathbf{S}^\top\mathbf{G})^+ \\ (\mathbf{S}^\top\mathbf{G})^+ \end{bmatrix} \boldsymbol{\Delta}_\mathbf{r}\mathbf{G}^\circ{}_{obs} \tag{16}$$

where $\mathbf{S}$ is the stoichiometric matrix of the training data-set, $\mathbf{G}$ is the group incidence matrix, and $\boldsymbol{\Delta}_\mathbf{r}\mathbf{G}^\circ{}_{obs}$ are the observed chemical Gibbs energies of the training set reactions. The $()^+$ sign represents the matrix pseudo-inverse. The orthogonal projections $\mathbf{P}_{\mathcal{R}(\mathbf{S})}$ and $\mathbf{P}_{\mathcal{N}(\mathbf{S}^\top)}$ are the projections on the range of $\mathbf{S}$ and the null-space of $\mathbf{S}^\top$ respectively. Note that orthogonal projection matrices satisfy the equations $\mathbf{P}^\top = \mathbf{P}$ and $\mathbf{P}^2 = \mathbf{P}$, and that $\mathbf{P}_{\mathcal{R}(\mathbf{S})} + \mathbf{P}_{\mathcal{N}(\mathbf{S}^\top)} = \mathbf{I}$.

For the covariance, we first define two parameters which represent the standard errors of the two sub-methods:

$$\begin{aligned} \alpha_{rc} &\equiv \frac{||\mathbf{e}_{rc}||}{\sqrt{n - \text{rank}(\mathbf{S})}} \\ \alpha_{gc} &\equiv \frac{||\mathbf{e}_{gc}||}{\sqrt{n - \text{rank}(\mathbf{S}^\top\mathbf{G})}}\,. \end{aligned} \tag{17}$$

The numerators ($||\mathbf{e}_{rc}||$ and $||\mathbf{e}_{gc}||$) are the total residual error of both regressions. Therefore, α_{rc} and α_{gc} are the unbiased estimators of the two standard errors (i.e. the uncertainties). We also define α_∞ as the prior uncertainty when there is no data at all about a compound or group. It should theoretically be set to infinity, but for numerical reasons we need to choose a finite value. Choosing a value which is too high might cause large floating-point rounding errors. On the other hand, a small value would underestimate the real uncertainty. In eQuilibrator, we set α_∞ to 10^5 kJ/mol by default, but that can easily be changed by the user.

Finally, we can define the following matrix:

$$\mathbf{L} \equiv \begin{bmatrix} \alpha_{rc}\ \mathbf{S}^+\mathbf{P}_{\mathcal{R}(\mathbf{S})} & \mathbf{0} \\ \alpha_{gc}\ (\mathbf{G}^\top\mathbf{S})^+\mathbf{G}^\top\mathbf{P}_{\mathcal{N}(\mathbf{S}^\top)} & \alpha_{gc}\ (\mathbf{G}^\top\mathbf{S})^+ \\ \alpha_\infty\ \mathbf{P}_{\mathcal{N}(\mathbf{S}^\top\mathbf{G})}\mathbf{G}^\top & \alpha_\infty\ \mathbf{P}_{\mathcal{N}(\mathbf{S}^\top\mathbf{G})} \end{bmatrix}^\top \tag{18}$$

The $\mathbf{L}$ matrix is constructed so that $\mathbf{L}\mathbf{L}^\top$ is the covariance matrix of the uncertainty of $\boldsymbol{\mu}$. For the full derivation of equation (18), see subsection S1.2.

Although $\mathbf{L}$ is a very wide matrix (with thousands of columns), we can greatly reduce its size by a rank revealing QR decomposition (also described in subsection sec:on-the-fly-derivation). We denote the reduced form by $\mathbf{L}_q$, where q is the rank of $\mathbf{L}$ and is also equal to the number of columns in $\mathbf{L}_q$. For the most recent eQuilibrator database $q = 669$.

S1.3.2 Estimation step

We wish to estimate the Gibbs energies of a new set of reactions, described by a stoichiometric matrix $\bar{\mathbf{X}}$. The top rows in this matrix correspond to known compounds that we had in the training set, while bottom rows represent new compounds. Let $\mathbf{G}'$ be the group incidence matrix of the new compounds. The estimate for the Gibbs energies of the reactions in $\bar{\mathbf{X}}$ will be:

$$\Delta_r \mathbf{G}^\circ(\bar{\mathbf{X}}) = \bar{\mathbf{X}}^\top \begin{bmatrix} \mathbf{I} & \mathbf{0} \\ \mathbf{0} & \mathbf{G}' \end{bmatrix} \boldsymbol{\mu} \tag{19}$$

and the square root of the covariance matrix will be:

$$\mathbf{Q}(\bar{\mathbf{X}}) = \bar{\mathbf{X}}^\top \begin{bmatrix} \mathbf{I} & \mathbf{0} \\ \mathbf{0} & \mathbf{G}' \end{bmatrix} \mathbf{L}_q \tag{20}$$

i.e. such that $\boldsymbol{\Sigma}(\mathbf{X}) = \mathbf{Q}(\bar{\mathbf{X}})\mathbf{Q}(\bar{\mathbf{X}})^\top$, see subsection S1.2 for details.

S1.4 Compressed storage of free energies

Let us consider a system independent from eQuilibrator that maintains a very large compound database or one that requires frequent updating. Theoretically, one could pre-calculate all possible formation energies and covariances (as explained in subsection S1.2) and redo that calculation every time a new compounds is added. However, this approach has two main limitations: (a) the size of the covariance matrix grows quadratically with the number of compounds, and (b) adding even one new compound requires complete recalculation of the covariance.

Here, we present for the first time a method to store a compressed version of the eQuilibrator database which can be used to generate the estimates and uncertainties using simple linear algebra (only matrix dot-products) and that grows linearly with the number of compounds both in terms of run-time and storage space.

First, we construct the $\mathbf{G}'$ matrix for all the new compounds, i.e., a collection of all of their group vectors. The mean estimates for the formation energies are:

$$\bar{\boldsymbol{\mu}} \equiv \begin{bmatrix} \mathbf{I} & \mathbf{0} \\ \mathbf{0} & \mathbf{G}' \end{bmatrix} \boldsymbol{\mu} \tag{21}$$

The uncertainties, however, require more attention. First, we note that only storing the uncertainty estimate of every formation energy by itself is not sufficient, since we cannot use them to calculate any off-diagonal value in the covariance matrix (see Figure S1). On the other hand, pre-calculating the full covariance matrix can require a prohibitive amount of memory. For example, for a database containing around one million compounds (which is the case for MetaNetX) the covariance matrix would occupy 8 terabytes.

Fortunately, the solution provided in equation (20), provides us with an opportunity. We define a new matrix:

$$\bar{\mathbf{L}}_q \equiv \begin{bmatrix} \mathbf{I} & \mathbf{0} \\ \mathbf{0} & \mathbf{G}' \end{bmatrix} \mathbf{L}_q \,. \tag{22}$$

$\bar{\mathbf{L}}_q$ is a constant matrix of size n by q, which amounts to only 2-3 gigabytes in our previous example. When we want to use our database to estimate the Gibbs energies of a set of reactions $\bar{\mathbf{X}}$, the distribution of the estimates will be given by a multivariate Gaussian with mean and standard deviation:

$$\begin{aligned} \Delta_r \mathbf{G}^\circ(\bar{\mathbf{X}}) &= \bar{\mathbf{X}}^\top \bar{\boldsymbol{\mu}} \\ \mathbf{Q}(\bar{\mathbf{X}}) &= \bar{\mathbf{X}}^\top \bar{\mathbf{L}}_q \\ \boldsymbol{\Sigma}(\mathbf{X}) &= \mathbf{Q}(\bar{\mathbf{X}})\mathbf{Q}(\bar{\mathbf{X}})^\top = \bar{\mathbf{X}}^\top \bar{\mathbf{L}}_q \bar{\mathbf{L}}_q^\top \bar{\mathbf{X}} \end{aligned} \tag{23}$$

Storing only $\bar{\boldsymbol{\mu}}$ and $\bar{\mathbf{L}}_q$ facilitates performing accurate thermodynamic calculations for a large number of compounds without requiring extremely large amounts of memory. Additionally, the pre-processing and estimation steps are decoupled, meaning that end-users do not need the entire eQuilibrator codebase and its dependencies. Furthermore, adding new compounds to the database is straightforward and does not require updating existing entries, but rather only augmenting $\bar{\boldsymbol{\mu}}$ and $\bar{\mathbf{L}}_q$ with the relevant data as extra rows.

S2 Sampling and optimization using the uncertainty covariance

The uncertainties of the free energies estimated with eQuilibrator are often correlated. Sometimes we have moieties whose formation energy has high uncertainty (such as coenzyme-A, Figure S1A), but this uncertainty cancels out in reactions where the moiety is present on both sides. In contrast, there are cases where reaction energies cannot be determined because of completely uncharacterized compounds (such as flavodoxin, Figure S1B), but using explicit formation energies reveals couplings between multiple reactions. Thus, it is often unclear whether one should use the domain of reaction energies or of formation energies. With eQuilibrator 3.0, we encourage the usage of the covariance matrix of the uncertainty when modeling multiple reactions. This matrix fully captures the correlations in the uncertainty of all quantities, and always constrains the values at least as much as when using independent uncertainties. In Figure S1, only using the covariance matrix allows to determine reaction directions in both examples. Importantly, the covariance can be used in the domains of formation as well as reaction energies without loss of information on the reaction energies. In this section we summarize how the convariance matrix can be used in sampling and constraint based methods.

Consider a reaction network with stoichiometric matrix $\bar{\mathbf{X}}$. The number of degrees of freedom $\bar{q}$ in the uncertainty is often smaller than the number of reactions n (note that $\bar{q} \leq q = 669$). Thus, it is convenient to represent the uncertainty with a random vector $\mathbf{m} \in \mathbb{R}^{\bar{q}}$ following the standard normal distribution $\mathcal{N}(\mathbf{0}, \mathbf{I})$ and a square root $\mathbf{Q}(\bar{\mathbf{X}}) \in \mathbb{R}^{n \times \bar{q}}$ of the covariance $\boldsymbol{\Sigma}(\mathbf{X})$ [3], such that:

$$\begin{aligned} \mathbf{m} &\sim \mathcal{N}(\mathbf{0}, \mathbf{I}) \\ \Delta_r \mathbf{G}'^\circ &= \Delta_r \mathbf{G}'^\circ(\bar{\mathbf{X}}) + \mathbf{Q}(\bar{\mathbf{X}})\mathbf{m} \ , \end{aligned} \tag{24}$$

where $\mathbf{I}$ is the $\bar{q}$-dimensional identity matrix. While $\mathbf{Q}(\bar{\mathbf{X}})$ can be computed from the eigenvalue decomposition of $\boldsymbol{\Sigma}(\mathbf{X})$, this is sensitive to numerical issues if $\bar{\mathbf{X}}$ is large. Instead, eQuilibrator computes $\mathbf{Q}(\bar{\mathbf{X}})$ directly as described in Section S1.2, providing a numerically accurate result.

In order to draw random samples of the Gibbs free energies we can first draw samples of $\mathbf{m}$ using standard methods and then compute the corresponding free energies using equation (24).

In a constraint-based setting, we can use the same formulation to define a quadratic constraint to bound free energies to a desired confidence level α:

$$\begin{aligned} ||\mathbf{m}||_2^2 &\leq \chi^2_{\bar{q};\alpha} \\ \Delta_r \mathbf{G}'^\circ &= \Delta_r \mathbf{G}'^\circ(\bar{\mathbf{X}}) + \mathbf{Q}(\bar{\mathbf{X}})\mathbf{m} \end{aligned} \tag{25}$$

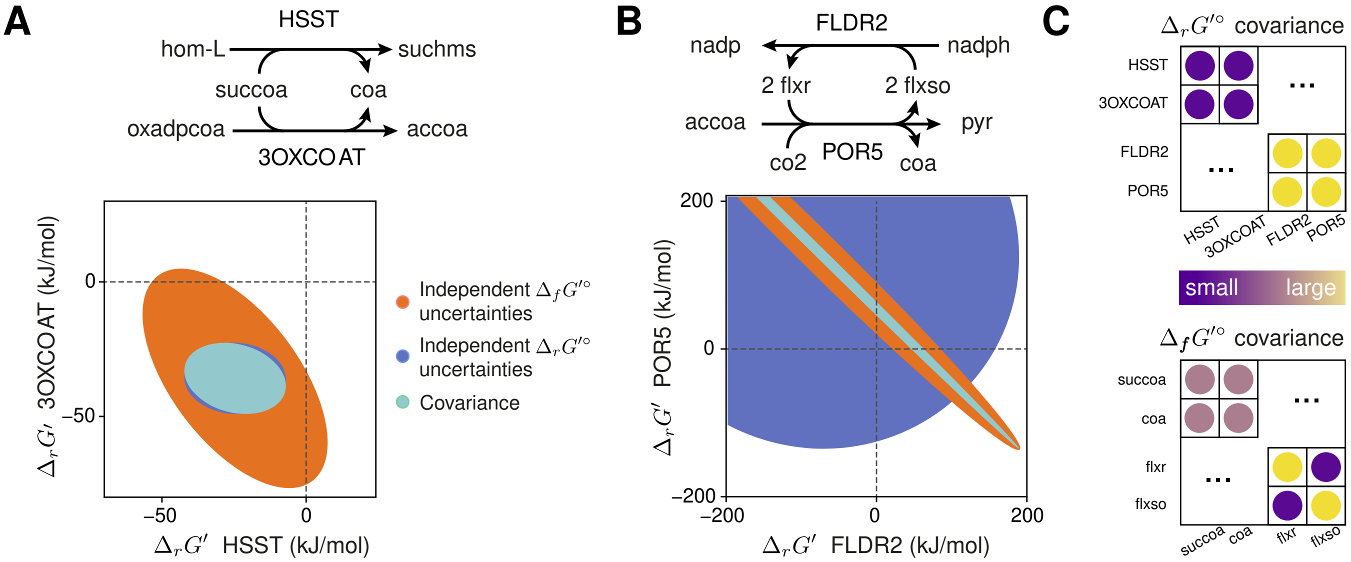

Figure S1: Examples for the importance of the covariance in the estimation uncertainty found in the iML1515 *E. coli* model. (**A**) Homoserine O-succinyltransferase (HSST) and 3-oxoadipyl-CoA thiolase (3OXCOAT) both convert succinyl-CoA (succoa) to CoA. Because of the uncertainty in the $\Delta_f G'^\circ$ of CoA, computing reaction energies from independent $\Delta_f G'^\circ$ estimates results in a large uncertainty (orange). As the $\Delta_f G'^\circ$ of succoa and CoA are strongly correlated, direct estimates of $\Delta_r G'^\circ$ have smaller uncertainty (blue) comparable to the uncertainty obtained using the covariance matrix of either $\Delta_f G'^\circ$ or $\Delta_r G'^\circ$ (cyan). (**B**) Pyruvate synthase (POR5) converts acetyl-CoA (accoa) into pyruvate (pyr) by oxidizing flavodoxin which, in iML1515, can be regenerated only through oxidation of NADPH (FLDR2). The $\Delta_r G'^\circ$ of both reactions is unknown. However, $\Delta_f G'^\circ$ of flxr and flxso must have the same value in both reactions, leading to strong correlation in the uncertainty of $\Delta_r G'^\circ$. Thus, *in-vivo* synthesis of pyruvate through POR5 is unfavorable. (**C**) Covariances of $\Delta_f G'^\circ$ and $\Delta_r G'^\circ$ yield the same information about reaction energies. The small uncertainty in $\Delta_r G'^\circ$ for HSST and 3OXCOAT matches the correlation in $\Delta_f G'^\circ$ of succoa and coa, while the coupling of FLDR2 and POR5 through flavodoxin is captured by the covariance in the $\Delta_r G'^\circ$ of the two reactions.

where $\chi^2_{\bar{q};\alpha}$ is the PPF (percent point function, or quantile function) of the χ^2-distribution with $\bar{q}$ degrees of freedom. In Python it can be calculated using `scipy.stats.chi2.ppf()`. One can find a code example for both constraint-based modeling and random sampling at the end of this section.

When quadratic constraints cannot be used, one can replace equation (25) with upper and lower bounds for each m_i separately, corresponding to a confidence interval α on each individual degree of freedom in the uncertainty:

$$|m_i| \leq \sqrt{\chi^2_{1;\alpha}} \qquad \forall\, 1 \leq i \leq \bar{q}\,. \tag{26}$$

Although simpler, this formulation should be used with care. Uncertainties are multivariate estimates and independent bounds can over-constrain the free energies, in particular for large networks. For example, when $\bar{q} = 50$ and $\alpha = 0.95$, the bounds in equation (26) define a confidence region on $\mathbf{m}$ with an overly restrictive confidence level $\alpha^{\bar{q}} = 0.08$.

```python
from equilibrator_api import ComponentContribution, Q_
import numpy, cvxpy, scipy.stats

# Define the reactions and calculate the mean and covariance
cc = ComponentContribution()
NTP3 = cc.parse_reaction_formula(
    "bigg.metabolite:gtp + bigg.metabolite:h2o = bigg.metabolite:gdp + bigg.metabolite:pi"
)
ADK3 = cc.parse_reaction_formula(
    "bigg.metabolite:adp + bigg.metabolite:gdp = bigg.metabolite:amp + bigg.metabolite:gtp"
)
reactions = [NTP3, ADK3]
standard_dgr_prime_mean, standard_dgr_cov = cc.standard_dg_prime_multi(
    reactions,
    uncertainty_representation="fullrank"
)

# Random sampling example
N = 1000
sampled_data = []
for i in range(N):
    m = numpy.random.randn(standard_dgr_cov.shape[1])
    standard_dgr_prime_sample = standard_dgr_prime_mean + standard_dgr_cov @ m
    sampled_data.append(standard_dgr_prime_sample.m_as("kJ/mol"))
    sampled_data = numpy.array(sampled_data)

# Constraint-based example (highest energy dissipation rate)
S = cc.create_stoichiometric_matrix(reactions)
alpha = 0.95
Nc, Nr = S.shape
Nq = standard_dgr_cov.shape[1]
lb, ub = 1e-4, 1e-2
ln_conc = cvxpy.Variable(shape=Nc, name="metabolite log concentration")
m = cvxpy.Variable(shape=Nq, name="covariance degrees of freedom")
constraints = [
    numpy.log(numpy.ones(Nc) * lb) <= ln_conc,  # lower bound on concentrations
    ln_conc <= numpy.log(numpy.ones(Nc) * ub),  # upper bound on concentrations
    cvxpy.norm2(m) <= scipy.stats.chi2.ppf(alpha, Nq) ** (0.5)  # quadratic bound on m based on CI
]
dg_prime = (
    standard_dgr_prime_mean.m_as("kJ/mol") +
    cc.RT.m_as("kJ/mol") * S.values.T @ ln_conc +
    standard_dgr_cov.m_as("kJ/mol") @ m
)
g_diss = dg_prime @ numpy.ones(Nr)
prob = cvxpy.Problem(cvxpy.Minimize(g_diss), constraints)
prob.solve()
print(prob.value)
```

S2.1 Gibbs energy balance

It has been recently proposed to use Gibbs energy balance to constrain thermodynamic models of metabolism [5, 7]. According to the notation in [5], we imagine a network composed by two sets of reactions – metabolic (MET) reactions describing metabolic conversions in the cell and/or metabolite transport, and exchange (EXG) reactions describing the transfer of metabolites across the system boundary. The fluxes of the exchange reactions (v_i) are defined such that they create a mass balance at steady state:

$$\forall i \in \text{EXG} \sum_{j \in \text{MET}} S_{ij} v_j = v_i \ . \tag{27}$$

For every exchange reaction, the Gibbs energy exchange rate is a product of the Gibbs energy of formation of the transferred metabolite and the associated flux, i.e. $\forall i \in \text{EXG} \ g_i = \Delta_f G'_i \ v_i$. For metabolic reactions, the Gibbs energy dissipation rate is the product of the Gibbs energy of reaction and the flux, i.e. $\forall j \in \text{MET} \ g_j = \Delta_r G'_j \ v_j$. The Gibbs energy balance constraint is meant to ensure that the sum of all Gibbs energy exchange rates must be equal to the sum of all Gibbs energy dissipation rates:

$$\sum_{i \in \text{EXG}} g_i = \sum_{j \in \text{MET}} g_j \ . \tag{28}$$

In vector form, the constraint can be rewritten as:

$$\begin{gathered} \mathbf{\Delta_f G'}^{\intercal}_{\text{EXG}} \cdot \mathbf{v}_{\text{EXG}} = \mathbf{\Delta_r G'}^{\intercal}_{\text{MET}} \cdot \mathbf{v}_{\text{MET}} \\ \begin{bmatrix} \mathbf{\Delta_f G'}_{\text{MET}} \\ \mathbf{\Delta_r G'}_{\text{EXG}} \end{bmatrix}^{\intercal} \cdot \begin{bmatrix} \mathbf{v}_{\text{MET}} \\ \mathbf{v}_{\text{EXG}} \end{bmatrix} = 0 \ . \end{gathered} \tag{29}$$

It has been argued that this constraint is redundant [2] in steady state networks, although the mathematical proof is incomplete. Here we confirm the hypothesis by showing that a steady state system always satisfies Gibbs energy balance, as long as consistent standard Gibbs energy estimates are used.

We consider a metabolic network with stoichiometric matrix **S**. The columns of **S** are ordered such that

$$\mathbf{S} = \begin{bmatrix} \mathbf{S}_{\text{MET}} & \mathbf{S}_{\text{EXG}} \end{bmatrix} \ , \tag{30}$$

where $\mathbf{S}_{\text{MET}}$ and $\mathbf{S}_{\text{EXG}}$ are the stoichiometric matrices of the metabolic and exchange reactions respectively. Assuming that the system is at steady state we have

$$\mathbf{S} \cdot \mathbf{v} = \begin{bmatrix} \mathbf{S}_{\text{MET}} & \mathbf{S}_{\text{EXG}} \end{bmatrix} \cdot \begin{bmatrix} \mathbf{v}_{\text{MET}} \\ \mathbf{v}_{\text{EXG}} \end{bmatrix} = 0 \ . \tag{31}$$

Since reaction energies represent differences of formation energies, any expression of reaction energies (including the common approach of accounting for transport thermodynamics through a correction term $\Delta_r G'^t$) [4] can be rewritten as a function of the formation energies. The covariance of the uncertainty provided by eQuilibrator guarantees that this always holds. Thus

$$\mathbf{\Delta_r G'} = \mathbf{S}^{\intercal}_{\text{MET}} \cdot \mathbf{\Delta_f G'} \ , \tag{32}$$

where $\mathbf{\Delta_f G'}$ is the vector of formation energies corrected for the properties of their respective compartment. We can augment equation 32 to map formation energies of exchanged metabolites to the respective exchange reactions as

$$\begin{bmatrix} \mathbf{\Delta_r G'}_{\text{MET}} \\ \mathbf{\Delta_f G'}_{\text{EXG}} \end{bmatrix} = \begin{bmatrix} \mathbf{S}^\top_{\text{MET}} \\ \mathbf{S}^\top_{\text{EXG}} \end{bmatrix} \cdot \mathbf{\Delta_f G'} \,. \tag{33}$$

We can now substitute equation 33 in 29, such that the Gibbs energy balance condition becomes:

$$\begin{aligned} \left(\begin{bmatrix} \mathbf{S}^\top_{\text{MET}} \\ \mathbf{S}^\top_{\text{EXG}} \end{bmatrix} \cdot \mathbf{\Delta_f G'} \right)^\top \cdot \begin{bmatrix} \mathbf{v}_{\text{MET}} \\ \mathbf{v}_{\text{EXG}} \end{bmatrix} = 0 \\ \mathbf{\Delta_f G'}^\top \cdot \begin{bmatrix} \mathbf{S}_{\text{MET}} & \mathbf{S}_{\text{EXG}} \end{bmatrix} \cdot \begin{bmatrix} \mathbf{v}_{\text{MET}} \\ \mathbf{v}_{\text{EXG}} \end{bmatrix} = 0 \,. \end{aligned} \tag{34}$$

However, equation 34 is a consequence of equation 31 and is always true if the system is at steady state. Thus, while the constraint in equation (28) successfully enforces the Gibbs energy balance when the uncertainties are uncorrelated, it does not provide additional constraints when the covariance of the uncertainty is available.

S3 Example code for multicompartmental reactions

In order to facilitate performing thermodynamic calculations for multi-compartment reactions, we created the `multicompartmental_standard_dg_prime` function which extends `standard_dg_prime` to reactions that traverse between two compartments (arbitrarily, we denote them *inner* and *outer*). In order to use this function one needs to create two reaction objects. In each reaction only the reactants (substrates and products) that are located on one of the compartments should be included (i.e. each one is a half-reaction). The other required arguments are the electrostatic potential difference ($\Delta\Phi$), and the pH level and ionic strength in the outer compartment. Note, that the pH and ionic strength of the inner compartment are simply the default values set by the `ComponentContribution` object.

One of the most difficult issues required for determining the Gibbs energy change of multi-compartment reactions, is knowing the charge of transported species. Here, we assume that the most abundance protonation level of each compound in the inner compartment, is also the one that is transported by the reaction. To change this default setting, protons can be added in order to increase or decrease the net charge (and number of protons).

This code example below shows how to estimate $\Delta_r G'^\circ$ for ATP synthase. This membrane complex converts proton motive force into energy stored in ATP. The full reaction is: ADP(c) + P_i(c) + n H^+(c) $\rightleftharpoons$ ATP(c) + H_2O(c) + n H^+(p), where (c) and (p) indicate the cytoplasmic and perisplasmic compartments, respectively, and n is the number of protons pumped. We set the aqueous conditions according to default values that should reflect the conditions in *E. coli*. The mean value of $\Delta_r G'^\circ$ can be plotted as a function of n (see figure S2). We show results for two alternative values of the eletrostatic potential ($\Delta\Phi$ equal to 140 mV or 170 mV) in order to demonstrate the dependence of the slope on $\Delta\Phi$. Since ATP synthase requires that the $\Delta_r G'^\circ < 0$ in order to create ATP, one can appreciate that when $\Delta\Phi$ is higher (170 mV) the minimum requirement for protons is 3, versus 4 when $\Delta\Phi$ is 140 mV.

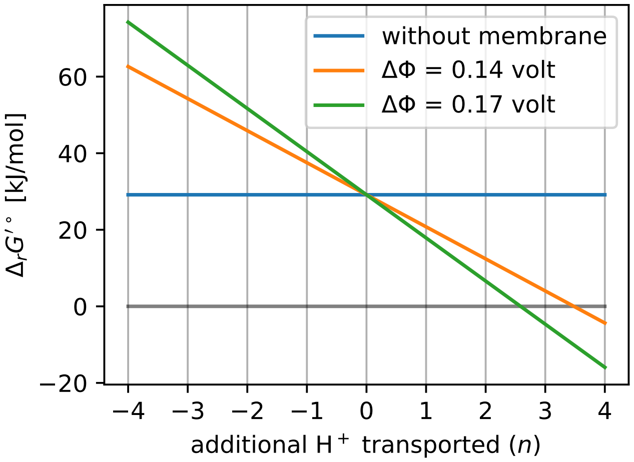

Figure S2: Example for the standard Gibbs energy of the ATP synthase reaction (ΔpH = 0.9, I_{cyt} = 250 mM, I_{per} = 200 mM, pMg = 3.0). Three conditions are compared: a single-compartment reaction, a multi-compartment reaction with $\Delta\Phi$ = 140 mV, and $\Delta\Phi$ = 170 mV. The x-axis represents the number of mole of protons that are pumped from the cytoplasm to the periplasm per mole of ATP generated.

References

[1] Tony F. Chan. Rank revealing QR factorizations. *Linear Algebra and its Applications*, 88-89:67–82, April 1987.

[2] Daan H De Groot, Julia Lischke, Riccardo Muolo, Robert Planqué, Frank J Bruggeman, and Bas Teusink. The common message of constraint-based optimization approaches: overflow metabolism is caused by two growth-limiting constraints. *Cellular and Molecular Life Sciences*, 77(3):441–453, 2020.

[3] Mattia G. Gollub, Hans-Michael Kaltenbach, and Jörg Stelling. Probabilistic thermodynamic analysis of metabolic networks. *Bioinformatics*, March 2021.

[4] H. S. Haraldsdóttir, I. Thiele, and R. M. T. Fleming. Quantitative Assignment of Reaction Directionality in a Multicompartmental Human Metabolic Reconstruction. *Biophysical Journal*, 102(8):1703–1711, April 2012.

[5] Bastian Niebel, Simeon Leupold, and Matthias Heinemann. An upper limit on Gibbs energy dissipation governs cellular metabolism. *Nature Metabolism*, 1(1):125, January 2019.

[6] Elad Noor, Hulda S. Haraldsdóttir, Ron Milo, and Ronan M. T. Fleming. Consistent Estimation of Gibbs Energy Using Component Contributions. *PLOS Computational Biology*, 9(7):e1003098, July 2013.

[7] Joana Saldida, Anna Paolo Muntoni, Daniele de Martino, Georg Hubmann, Bastian Niebel, A. Mareike Schmidt, Alfredo Braunstein, Andreas Milias-Argeits, and Matthias Heinemann. Unbiased metabolic flux inference through combined thermodynamic and 13C flux analysis. *bioRxiv*, page 2020.06.29.177063, June 2020. Publisher: Cold Spring Harbor Laboratory Section: New Results.

```
from equilibrator_api import ComponentContribution, Q_
cc = ComponentContribution()

data_series = []
PMF_RANGE = 4
for phi in [0.14, 0.17]:
    e_potential_difference = Q_(phi, "V")
    cytoplasmic_p_h = Q_(7.4)
    cytoplasmic_ionic_strength = Q_("250 mM")
    cytoplasmic_p_mg = Q_(3.0)
    periplasmic_p_h = Q_(6.5)
    periplasmic_ionic_strength = Q_("200 mM")
    periplasmic_p_mg = Q_(3.0)
    data = []
    for n_pmf in numpy.arange(-PMF_RANGE, PMF_RANGE+1):
        cytoplasmic_reaction = (
            f"bigg.metabolite:adp + bigg.metabolite:pi + {n_pmf} bigg.metabolite:h = "
            "bigg.metabolite:h2o + bigg.metabolite:atp"
        )
        periplasmic_reaction = f" = {n_pmf} bigg.metabolite:h"
        cc.p_h = cytoplasmic_p_h
        cc.ionic_strength = cytoplasmic_ionic_strength
        cc.p_mg = cytoplasmic_p_mg
        standard_dg_prime = cc.multicompartmental_standard_dg_prime(
            reaction_inner=cc.parse_reaction_formula(cytoplasmic_reaction),
            reaction_outer=cc.parse_reaction_formula(periplasmic_reaction),
            e_potential_difference=e_potential_difference,
            p_h_outer=periplasmic_p_h,
            ionic_strength_outer=periplasmic_ionic_strength,
            p_mg_outer=periplasmic_p_mg
        )
        data.append((n_pmf, standard_dg_prime.value.m_as("kJ/mol")))
        pmf, dg = zip(*data)
        data_series.append((
            e_potential_difference, cytoplasmic_p_h, periplasmic_p_h, pmf, dg
        ))
fig, ax = plt.subplots(1, 1, figsize=(3, 3), dpi=150, sharey=True, sharex=True)
ax.plot([-PMF_RANGE, PMF_RANGE], [0, 0], "k-", alpha=0.3)
for e_potential_difference, _, _, pmf, dg in data_series:
    ax.plot(
        pmf, dg,
        label=f"$\Phi$ = {e_potential_difference}"
    )
ax.set_xlabel("additional H$^+$ transported")
ax.set_ylabel("$\Delta_r G'^\circ$ [kJ/mol]")
ax.legend(loc="best")
```
